# Biomimic Vein-Like Transparent Conducting Electrodes with Low Sheet Resistance and Metal Consumption

**DOI:** 10.1007/s40820-019-0359-9

**Published:** 2020-01-10

**Authors:** Guobin Jia, Jonathan Plentz, Andrea Dellith, Christa Schmidt, Jan Dellith, Gabriele Schmidl, Gudrun Andrä

**Affiliations:** Leibniz Institute of Photonic Technology (Leibniz IPHT), Albert-Einstein-Str. 9, 07745 Jena, Germany

**Keywords:** Biomimic leaf vein network, Transparent conducting electrode, Sheet resistance, Metal consumption

## Abstract

In this contribution, inspired by the excellent resource management and material transport function of leaf veins, the electrical transport function of metallized leaf veins is mimicked from the material transport function of the vein networks. By electroless copper plating on real leaf vein networks with copper thickness of only several hundred nanometre up to several micrometre, certain leaf veins can be converted to transparent conductive electrodes with an ultralow sheet resistance 100 times lower than that of state-of-the-art indium tin oxide thin films, combined with a broadband optical transmission of above 80% in the UV–VIS–IR range. Additionally, the resource efficiency of the vein-like electrode is characterized by the small amount of material needed to build up the networks and the low copper consumption during metallization. In particular, the high current density transport capability of the electrode of > 6000 A cm^−2^ was demonstrated. These superior properties of the vein-like structures inspire the design of high-performance transparent conductive electrodes without using critical materials and may significantly reduce the Ag consumption down to < 10% of the current level for mass production of solar cells and will contribute greatly to the electrode for high power density concentrator solar cells, high power density Li-ion batteries, and supercapacitors.

**Electronic supplementary material:**

The online version of this article (10.1007/s40820-019-0359-9) contains supplementary material, which is available to authorized users.

## Highlights

- The electrical transport of the metallized vein networks is mimicked from the material transport function of the leaf vein networks.
- The vein-like transparent conducting electrodes show ultralow sheet resistance < 0.1 Ω □^−1^, broadband optical transparency > 80%, and high current density transport capability > 6000 A cm^−2^.
- The metal consumption for the metallization of the leaf veins can be as low as 4 g m^−2^.

**Electronic supplementary material:**

The online version of this article (10.1007/s40820-019-0359-9) contains supplementary material, which is available to authorized users.

## Graphical abstract

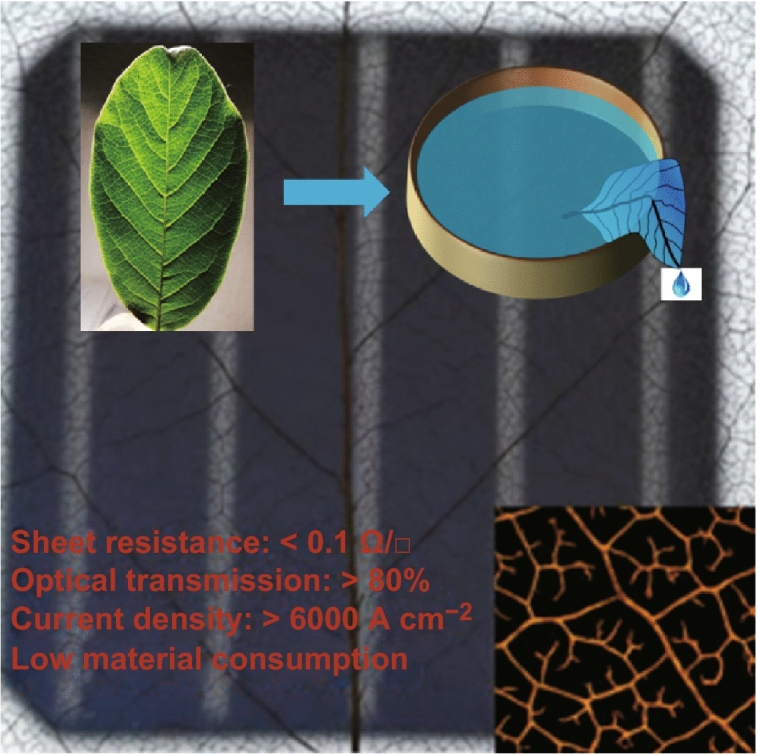

## Introduction

Plant leaves behave like photochemical factories that convert water and CO_2_ into carbohydrates and oxygen by photosynthesis. The principle of photosynthesis, i.e. harvesting of sunlight by the chlorophyll inside the leaves, has been mimicked by many artificial devices, such as solar cells [1, 2], which transform photon energy directly to electricity or to stored chemical energy by photochemical cells [3–5].

**Fig. 1:**
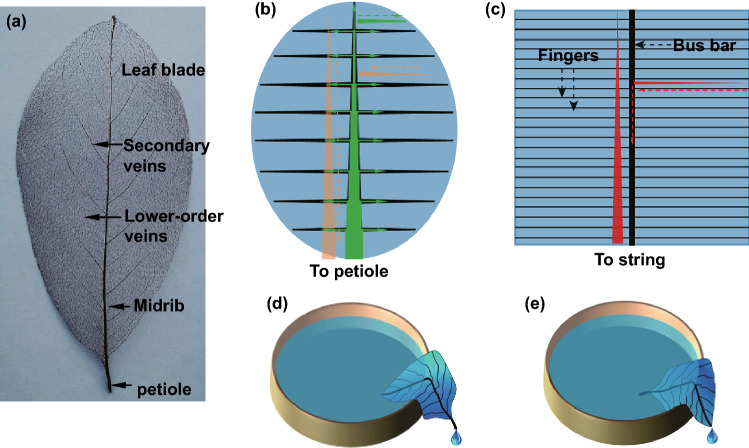
**a** Architecture of a *Magnolia liliiflora* leaf vein network. **b** Scheme illustrating the bidirectional transport process in the leaf veins. **c** Current collection in solar cells by the front grid. **d** Demonstration of water transport from the blade (immersed in water) to the midrib through the capillaries and subsequently drops down outside the Petri dish at the petiole (Video S1). **e** Water is collected from the petiole to the midrib, transported through the capillaries in the midrib to the leaf tip, and drops down (Video S2)

The leaf vein networks [6, 7] (example of a Magnolia liliiflora leaf vein is shown in Fig. 1a) play an important role in the photosynthesis process, and they supply water and nutrients to the leaf cells and transport away the photosynthesized carbohydrates to other parts of the plants for their growth. Additionally, they serve as a flexible backbone for the mechanical stability of the leaves [8, 9]. To fulfil the multifunction, plants have to efficiently use the resources needed for building up the vein network, ensure the bidirectional transport function while providing sufficient mechanical stability [9]. In addition, the vein networks are able to manage the great variation in transportation needed during the day and night as well as during different seasons.

Especially, the transport function of the leaf vein networks [9] shares many features in common with the electrodes used in solar cells, photochemical cells, and energy storage devices such as Li-ion batteries and supercapacitors [10], and many other optoelectronic devices such as light emitting diodes (LEDs) and photo detectors, through which electrical current or signals have to be collected or distributed.

Most leaf veins have hierarchical structures, including the petiole, midrib, and secondary and lower-order veins, as shown in Fig. 1a. A gradual thickening of the midrib towards the petiole as well as the secondary veins towards the midrib is the typical architecture, which reflects the variation in the amount of the resources transported along the veins and the midrib (Fig. 1b). The supply of water and nutrients (green arrows in Fig. 1b) attenuates from the petiole towards the midrib and subsequently to the leaf blade. The generated carbohydrates are transported in the opposite direction but with increasing amounts (orange arrow in Fig. 1b). The lower-order thin veins connect the midrib and the secondary veins, forming an interconnected network. Such a structure ideally serves for the bidirectional transport to and from the leaf cells, ensuring that the materials are collected and distributed uniformly throughout the whole blade. The vein networks retain the function even if parts of them are damaged, as known from nature and shown in the images in Fig. S1.

In the case of solar cells, the front electrode collects the generated photocurrent and transports it towards the metal fingers to the bus bars and subsequently to the strings as shown in Fig. 1c. An increase in the current density (*J*) along the collection direction is expected, as illustrated in Fig. 1c by the red arrows along the grid fingers as well as the bus bar. However, for simplification of technical fabrication, the thicknesses of the fingers and the bus bars are typically kept uniform (Fig. 1c) in most of the comb-like electrodes. This geometry does not reflect the variation in *J* along the fingers and the bus bars. The present comb-like configurations have a negative impact on resource efficiency since unnecessarily thick silver fingers have to be fabricated near the edge of solar cells where *J* is still low, leading to high silver consumption, high shadowing of the active area, and high cost of the solar panels. In comparison with the leaf vein network architectures, the H-comb metal grid features errors such as breaks that will lead to severe degradation of the performance of the solar cells. The bidirectional transport function of a leaf vein is illustrated in Fig. 1d, e, and the corresponding Video S1 demonstrates water transport from blade (immersed in water) to the midrib and subsequently drops down outside the Petri dish at the petiole, and Video S2 demonstrates water transport in the opposite direction, i.e. the leaf vein is placed in the opposite direction so that the water is collected from the petiole to the midrib, and transported through the capillaries in the midrib to the leaf tip and drops down.

In this contribution, inspired by the transport function and the excellent resource management of the leaf vein networks, we mimic their transport function in terms of electrical current flow through metallized veins realized by electroless copper plating (ECP) nano- and micro-technology [11]. It is found that the metallized vein-like architectures can be used as very resource-efficient transparent conductive electrodes (TCEs) with a sheet resistance (*R*_sh_) two orders of magnitude lower than that of state-of-the-art indium tin oxide (ITO) films and with high broadband optical transmission (*T*) [12], and to our knowledge, these vein-like TCEs present the lowest sheet resistance and comparable optical transparency to previous works so far. Especially, such vein-like TCEs can be used to replace ITO and saving the critical material indium and significantly reduce the noble metal consumption for electrode applications. Particularly, high current density transport property has been demonstrated, a *J* of more than 6000 A cm^−2^ can be transported through the metallized vein-like networks.

## Experimental Sections

### Preparation of Leaf Veins

A 500 mL water solution containing of 50 g NaOH (Sigma-Aldrich) and 25 g Na_2_CO_3_ (Sigma-Aldrich) in a glass cup was heated to 90 °C. Then, the freshly picked or dried *Magnolia liliiflora*, *Populus* × *canescens, or Fagus sylvatica* leaves were treated in the bath for 30 min. Subsequently, 20 mL H_2_O_2_ (30%) was added and treated for another 15 min. Then, the cooked leaves were put on Petri dish and washed with water one by one, and the tissues of the leaves were gently brushed away. The veins were subsequently put in a water bath and sonicated to remove the residue on the veins, leaving behind the clean veins. The leaf veins were further bleached by 10% H_2_O_2_ water solution for 30 min and then thoroughly rinsed with deionized (DI) water. The veins were then sandwiched in a book and pressed for flattening and drying for at least 2 days. Most of the results have been obtained using *Magnolia liliiflora* leaf veins (MLLVs), since they have moderate vein density. MLLVs have a balanced optical transmission (lower density is preferred) and conductivity (high density is preferred).

The mass of the leaf veins (as well as for the Cu-coated veins) was measured by a Sartorius Practum64-1S precision balance. Each leaf vein was measured before and after the electroless plating (after complete drying).

The area of the leaf vein is determined by a procedure described in Section S3.

### Electroless Copper Plating

The great advantage of the electroless copper plating (ECP) is that it can be performed on non-conducting substrates such as ceramics, polymer, or biomaterials. This is the case in this work, where a copper thin film was realized on the non-conductive vein networks. The ECP is performed in a three-step process after the formula in Ref. [11] with slight modifications.

#### Sensitization of the Leaf Veins

The sensitization bath contains 5 g L^−1^ SnCl_2_ (Sigma-Aldrich) added with 40 mL L^−1^ of 38% HCl (J.T. Baker). The veins were dipped into the sensitization solution one by one, and each keeps in the solution for 5 min. Then, they were rinsed twice with DI water.

#### Activation by Pd Catalyst Deposition

The veins were subsequently put into the activation bath containing 0.25 g L^−1^ PdCl_2_ (Sigma-Aldrich) and 2.5 mL L^−1^ of 38% HCl (J.T. Baker) for 5 min one by one. In this step, the Pd^2+^ ions are reduced to Pd nanoparticles and deposited on the vein surface, while the Sn^2+^ ions are oxidized to Sn^4+^. The Pd nanoparticles serve as the nucleus for the ECP. The reaction during the activation process is as Eq. (1):

1 $${\text{Pd}}^{2 + } + {\text{Sn}}^{2 + } \to {\text{Pd}} + {\text{Sn}}^{4 + }$$

After the activation process, the veins were thoroughly rinsed with DI water several times.

#### Electroless copper plating bath

The above-treated veins were placed in a plating bath containing 10 g L^−1^ copper sulphate pentahydrate (CuSO_4_·5H_2_O), 10 g L^−1^ sodium hydroxide (NaOH), 50 g L^−1^ of potassium sodium tartrate (KNaC_4_H_4_O_6_·4H_2_O), and 15 ml L^−1^ formaldehyde (CHOH). The CHOH serves as the reducing agent for the reduction in Cu^2+^ ions to Cu. A Cu thin film grows auto-catalytically on the veins.

The as-plated leaf veins were then rinsed several times with DI water and sandwiched in a book between two papers for flattening and drying for several days (at least for 2 days for complete drying).

The thickness of the Cu layer on the vein has been estimated with the help of the cross-sectional SEM image described in Section S4.

An annealing procedure is described in Section S5.

#### Electroplating of Ag on the Conducting Cu-plated Veins

The Cu-plated veins can be covered with different metals and alloys to achieve the desired electrical, anti-corrosion, solderability, aesthetic properties of the network, for example plating of metals with different work functions for the applications as electrodes, coating with protective Ni layer to improve the anti-corrosion, plating with low melting temperature metals or alloys to tune the solderability of the veins, with Ag or Au coating to decorate the veins or for the integration in optoelectrodevices.

The veins were converted to highly conducting networks by the ECP. Afterwards, a great variety of metals and alloys can be plated around the veins by common electroplating technologies. This will encapsulate the copper and form a coaxial metal/copper/vein structure. In this work, Ag electroplating on top of the Cu-coated veins has been performed as an example.

Ag electroplating was performed by using 100 mL 0.1% (weight ratio) AgNO_3_ (J. T. Baker) water solution with addition of 1 mL HNO_3_ (65% from Chemsolute) at an applied voltage of 1.5 V. A silver wire serves as anode and the Cu-plated veins as cathode. The plating time was 30 s. After the Ag electroplating, the samples were immersed in deionized water for 5 min and the water was discarded afterwards. This procedure was repeatedly performed to clean any residue chemicals.

#### Bending Tests

The flexibility of the vein-like electrodes was investigated by carefully designed bending tests, during which the resistance was measured by a precision multimeter with an accuracy of 1 mΩ (ISO-TECH, LCR-1701). Because the copper-plated MLLVs have very small resistance below 0.5 Ω, any potential error sources of contact resistance have to be excluded. In this circumstance, all the connections outside the multimeter were made by permanent soldering, including the copper-plated MLLV soldered between two pieces of circuit boards, so that reliable measurements of the resistance can be taken during the bending tests. The bending radius can be varied by using isolating glass tubes or rods with radii of 5.35, 3.5, and 2.75 mm, respectively.

## Results and Discussion

### Ultralow Electrical Sheet Resistance with High Broadband Optical Transmission

**Fig. 2:**
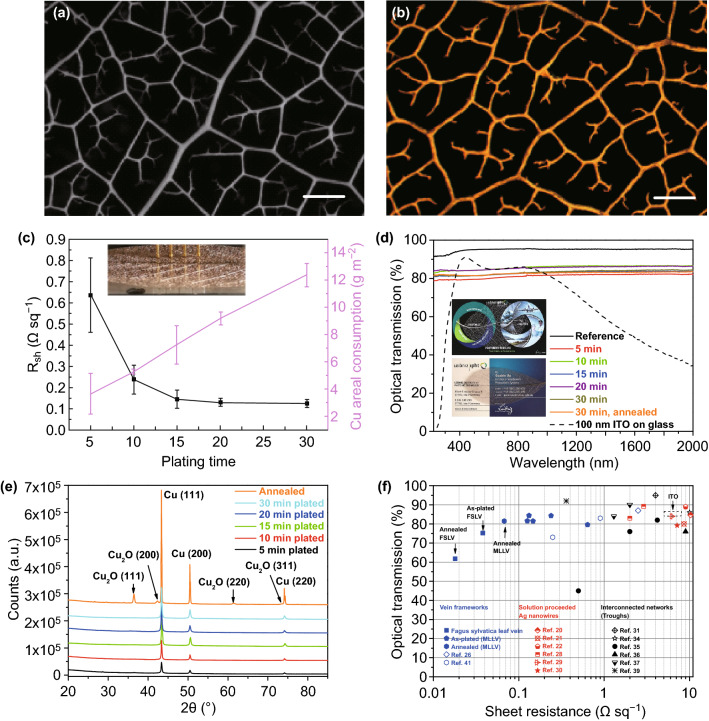
**a** A micrograph of *Magnolia liliiflora* leaf vein (MLLV). **b** A micrograph of the 30 min electroless Cu-plated MLLV; scale bars for both a and b: 500 µm. **c** The *R*_sh_ (black) and Cu consumption on the veins (magenta) versus plating time. **d** Optical transmission of the pure and as-plated veins with different plating times and after annealing. A transmission spectrum of 100 nm ITO deposited on 0.7-mm Borofloat glass for comparison. **e** XRD measurements of the as-plated and annealed Cu layers. **f** A summary of the recently developed TCEs based on perforated networks with a *R*_sh_ of below 10 Ω □^−1^ and an optical transmission (at 550 nm) of around 80%

*Magnolia liliiflora* leaf veins (MLLVs) have been chosen from more than 30 different leaves as frameworks for the ECP in this work. MLLVs have a medium vein density (Fig. 2a) and show a balanced optical transmission (lower density is preferred) and electrical conductivity (higher density is preferred) after ECP (Fig. 2b). An estimation of the transmission can be made from both micrographs, in which the relation of black area to total area gives the transmission. In both cases, optical transmission (*T*) is approximately 85% and fits well with the optical transmission data measured experimentally.

The electrical sheet resistance (*R*_sh_) versus plating time is demonstrated in Fig. 2c, where the inset shows an image of Cu-plated MLLVs during the four-probe measurement. The Cu-plated MLLVs have an average *R*_sh_ of 0.640 Ω □^−1^ and a deviation of 27.6% even for a short plating time of 5 min. *R*_sh_ as well as the deviation decreases strongly with the plating time, and an ultralow *R*_sh_ of 0.126 Ω □^−1^ is reached (with only 15.1% deviation) at a plating time of 30 min. Copper thicknesses of 0.5 and ~ 5.5 µm have been determined in the two cases (Section S4).

The optical transmission (*T*) of the pure veins (taken as a reference) can be as high as 91.5% in the UV (220–380 nm) range, above 94% in the violet (380–450 nm) range, and above 95% in the VIS to IR (450–2000 nm) range with a value of 95.1% at 550 nm. The *T* of 91.5% at 220 nm is related to the “intrinsic” transparency of the perforation of MLLV since the UV photons may not penetrate through the vein stems and can only pass through the perforations. The increase in the transmission from 300 to 2000 nm is mainly related to an additional contribution from photons penetrating through the semi-transparent veins. Upon ECP, the veins become opaque, and light can only go through the perforation. The highly conductive veins show a high broadband *T* of up to 84.6% at 550 nm. *T* is almost constant over the entire measurement range, as shown in Fig. 2d. It is noted that there is no clear correlation between the plating time and *T*, which is due to the variation in individual vein structures. For comparison, a transmission spectrum of a 100 nm ITO thin film deposited on 0.7-mm Borofloat glass (deposited by magnetron sputtering and subsequently post-annealed at 250 °C with an *R*_sh_ of 34 Ω □^−1^ at *T*_550 nm_ = 86.1%) is attached (black dashed line). It is demonstrated that the ITO thin film has slightly better *T* in the range of 380–1000 nm in certain cases, but the vein-based TCEs have much better *T* below 380 nm and above 1000 nm, with *R*_sh_ more than two orders of magnitude better than that of the ITO thin film.

By annealing of the pure vein and the 30 min Cu-plated veins in an inert N_2_ atmosphere up to 970 °C, the pure vein can be converted to a conducting carbon network (Section S5). The carbonized vein networks are very brittle and usually break during a four-point probe measurement; thus, it does not allow systematic measurements. Nevertheless, we compared the resistance measured on the as-plated veins with known *R*_sh_ values and estimated the *R*_sh_ of the carbon network to be approximately 100 Ω □^−1^.

Surprisingly, mechanically stable networks can be obtained with the Cu-plated veins after annealing (Section S5). The annealing process reduces the *R*_sh_ by 50% down to an ultralow value of 0.068 Ω □^−1^ with a *T* of 81.5%. Therefore, the *R*_sh_ is almost two orders of magnitude lower (but with higher broadband *T*) than that of state-of-the-art ITO films with an *R*_sh_ of 5–8 Ω □^−1^ and a *T* of 85% [12]. It is noted that on *Fagus sylvatica* leaf veins (FSLVs) with higher vein density than the MLLVs, a *R*_sh_ of 0.038 Ω □^−1^ with *T *= 75.2% for the 30 min as-plated sample can be obtained, and an exceptionally low *R*_sh_ of 0.018 Ω □^−1^ with average *T *= 61.8% for the annealed sample have been achieved (Section S6).

### Resource Efficiency

The most prominent features are the resource efficiency of the veins and of the Cu-plated veins. The average areal density of the MLLVs determined in this work is only 5.32 ± 1.27 g m^−2^. This value is approximately 15 times lower than that of the commonly used 80 gsm (grams per square metre) print paper. The average Cu consumption on the veins is between 3.68 g m^−2^ (5 min) and 12.40 g m^−2^ (30 min), depending on the plating time (Fig. 2c), and these values are equivalent to 0.41 and 1.38 µm if the copper is homogeneously deposited on the area, respectively. These quantities correspond to 0.09–0.30 g per cell if the TCEs would be used for solar cells with a standard size of 15.6 × 15.6 cm^2^. The Cu consumption of the 5 min plated veins is at the same level as the Ag consumption for solar cells [12], and the *R*_sh_ of 0.640 Ω □^−1^ is also comparable with that of the Ag-based electrode of a commercial solar cell with 0.6–0.7 Ω □^−1^. However, Cu is approximately 80 times cheaper than Ag, which will significantly reduce the cost of metallization.

The Cu grown on the veins during ECP forms a compact layer even within 5 min. The average crystal sizes of the as-plated samples are in the range of 45–75 nm, as determined by the X-ray diffraction (XRD) patterns shown in Fig. 2e. A substantial improvement in the crystallinity and growth of larger crystallites up to 140 nm was observed after thermal annealing at 970 °C. This effect was confirmed by SEM images of the annealed sample, which shows large crystals with clear facets (Section S7). The improved crystallinity explains the decrease in *R*_sh_ upon annealing.

The Cu-plated veins represent a kind of “perforated” transparent conductive electrode (TCE). In recent years, tremendous effort has been made to develop electrodes with an ultralow *R*_sh_ combined with a high *T*. This objective was mainly motivated by the search for alternatives to replace the widely used ITO-based TCEs with a rather high *R*_sh_ of 5–8 Ω □^−1^ and *T *= 85% [12] and simultaneously reduce the critical raw material like indium, since its availability could be a major issue in the near future. Additionally, ITO is quite brittle, which limits its applications in flexible optoelectronic devices. Furthermore, ITO-based TCEs work well only around the visible wavelength range and do not perform well in the UV and near-infrared ranges due to strong absorption loss [13] and high reflection loss [14], respectively. Therefore, ITO is inappropriate for many optoelectronic applications, such as light emitting diodes (LEDs) working in the UV [15, 16] and infrared range [17] or multicolour LEDs [18]. The main drawback of thin film-based TCEs is that the electrical conductivity and *T* are coupled together and competing with each other. That means, to achieve the desired higher conductivity, a thicker film is needed, which will lower *T*, and vice versa. In the perforated leaf vein-based TCEs, the conductivity and *T* are only weakly related. The conductivity can simply be improved by increasing the thickness of the metal layer without substantially reducing *T*, which is a great advantage in the designing of electrodes for many applications.

For many applications, such as large-area solar cells, highly efficient tandem solar cells, large-area displays, concentrating solar cells [19], or electrodes used in Li-ion batteries and supercapacitors, a much lower *R*_sh_ is necessary to reduce the series resistance and the power loss of the devices. In particular, electrodes with the combination of a low *R*_sh_ and a high *T* are crucial for reducing the resistive and shading losses in photovoltaic (PV) applications.

Perforated TCEs are a promising route to fulfil the requirements for the above-mentioned applications and therefore have been intensively investigated recently. Solution-processed Ag nanowire networks [20–31], Cu nanowire networks [32], lithographically fabricated metal meshes [33], and some novel approaches using cracked polymers [34], cracked TiO_2_ thin layers [35], In_2_O_3_ grain boundary lithography [36], cracked silica nanoparticle thin layers [37], and nanotrough [38] have been proposed. The published results for *R*_sh_ values below 10 Ω □^−1^ combined with *T* values of approximately 80% along with those obtained in this work are summarized in Fig. 2f, in which the black symbols are those TCEs obtained with interconnected metal networks, and the red symbols are those obtained with solution-processed (including post-treatments) Ag nanowire thin films. The blue hollow symbols present the previous work performed using leaf veins as frameworks. The results in this work are presented as solid blue symbols. The *R*_sh_ and optical transmission of the state-of-the-art ITO film are included as dashed squares for comparison. In most cases, *R*_*sh*_ of 1–10 Ω □^−1^ with a *T* around 80% can be reached with these novel approaches. In one case, Hsu et al. [39] reached an excellent low *R*_sh_ of 0.36 Ω □^−1^ with a *T* of 92% using a hybrid approach between Au nanowires and Cu mesowires.

It is noted that two groups have fabricated TCEs using leaf veins as frameworks. Han et al. [40] obtained metallized veins by sputtering of an Ag thin layer. They reached an *R*_sh_ of approximately 2.5 Ω □^−1^, which is two times better than that of the state-of-the-art ITO but with the same *T *= 85% [12]. In Han’s work, the metallization was performed by sputtering of Ag in a vacuum chamber, and obviously Ag cannot be coated on the shadowed side, and the sputtered Ag should have amorphous nature, so the “sheet resistance” is poor. Yu et al. [41] used polymer-assisted metal deposition to convert leaf veins to TCEs. An *R*_sh_ of 0.9 Ω □^−1^ combined with a *T* of ~ 83% was reached. For a higher vein density, a lower *R*_sh_ of 0.25 Ω □^−1^ but with a *T* of only 73% was reached. In Yu’s work, polymer was involved, which might be responsible for the copper layer delamination in certain cases. In our cases, the electroless copper plating takes place directly on the Pd seed layer anchored on the vein surface, and the copper layer has a strong adhesion; a delamination of the copper layer have never been observed during the bending tests, cutting for the cross-sectional SEM investigation, during the soldering (soldering temperature 350 °C) and storage over one year.

These two papers demonstrated some of the interesting properties of the vein-like electrodes; however, the full potential of the vein-like electrodes has not been demonstrated; particularly, the combined *R*_sh_ and optical transparency is still insufficient to attract the attention of the PV community, which require electrodes to have optical transparency of > 80% combined with *R*_sh_ < 0.5 Ω □^−1^. Especially, there was no information about the metal consumption and the high current density transport capability given in the papers, which are great features of the vein-like electrodes what we found in this contribution. These two features closely relate to the resource efficiency, which become more and more a key issue for a sustainable development of large-scale production of PV modules, and the breakdown of the PV costs to make it competitive to fossil energy sources.

Silver has been chosen as the most commonly used electrode material for PV due to its high conductivity. With the increase in large volume production of PV modules, Ag consumption has become a major issue, leading to a short supply of the noble metal, increase in price, and a slowdown of the cost reduction to the solar modules. The average Ag consumption was approximately 90 mg for standard solar cells with an area of 15.6 × 15.6 cm^2^ and accounted for 13% of the solar cell price in 2017, according to the “International Technology Roadmap for Photovoltaic (ITRPV)” [42]. The target is to reduce the silver content down to 50 mg per solar cell in 2028, which could be achieved by fully or partly replacing Ag with another earth-abundant and cheap metal. Copper is ~ 80 times cheaper with a conductivity only 6% lower than Ag and could be promising alternative for the PV applications. However, the great issues in copper-based electrodes are that it introduces deep-level defect states in silicon [43]. Great concern arises that the PV performance could be degraded by Cu-related defects, which are caused by the diffusion of Cu ions released from the electrode during operation.

For the potential application of Cu-plated vein-like TCEs to silicon solar cells, the issue with Cu contamination has to be addressed. We demonstrate in the following experiment that Ag can be deposited on top of the Cu layer by common electroplating technology, since the highly conducting veins allow apply electric current through an Ag-containing electrolyte. Such an encapsulation coating can be used to seal the Cu layer, preventing the release of Cu ions and thus the degradation of the solar cells. Encapsulation of other metals or alloys can be performed as well by appropriate electroplating processes, so that the electric properties, such as the conductivity and work function, can be tuned, and the anti-corrosion, solderability, and aesthetic properties of the networks can be improved.

**Fig. 3:**
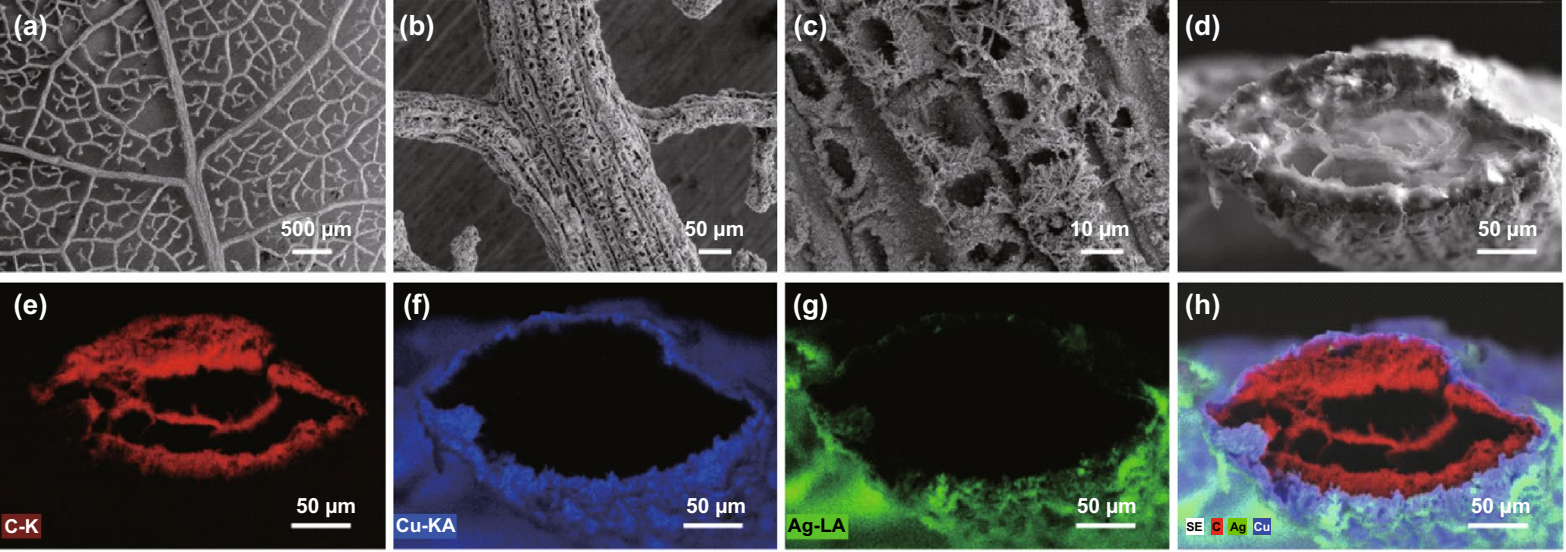
Encapsulation by electroplating of Ag on Cu-plated veins. **a** SEM images of electroplated Ag on a Cu-plated *Populus *×* canescens* leaf vein network. **b** Details on the midrib. **c** Ag dendrites grown on the midrib. **d** Secondary electron (SE) image at the cross section of the midrib. **e** EDX mapping of the carbon Kα-line at the cross section of the midrib. **f** EDX mapping of the Cu Kα line. **g** EDX mapping of the Ag Lα line. **h** Mixed colour EDX mapping of the three elements and the SE image

An example of Ag electroplating on Cu is demonstrated in the SEM images in Fig. 3. A 30 min ECP of Cu and an only 30 s electroplated Ag thin layer on *Populus* × *canescens* leaf veins forming an Ag/Cu/vein coaxial network. The 30 s Ag plating process is enough to fully encapsulate the Cu-plated vein. The amount of Ag consumed is only 0.5 mg over a leaf area of 23 cm^2^. Therefore, the Ag consumption is only 220 mg m^−2^ and equivalent to 5.3 mg per solar cell. This value is less than 6% of the present level (90 mg) and is only 10.6% of the goal (50 mg) set for the roadmap for PV in 2028 [42].

### High Current Density Transport Capability

It is noted that all the metallized veins plated with different times can sustain the applied current of 100 mA during the four-point probe measurements without any visible changes or damage. A conservative estimation shows that a high *J* up to 6329 A cm^−2^ can pass locally through the thin veins (Section S8).

The high current density transport function of the leaf vein network could be a result of the network architectures, so that the current can be distributed over the whole network. Furthermore, the high-quality copper crystallites (with larger crystal size of ~ 45–75 nm) are grown directly on the vein framework, and this low temperature process has great advantage for the good conductivity compared to Ag screen or inkjet printing technologies, where the Ag nanoparticles (~ 10 nm) have to be sintered at high temperature under inert or forming gas ambient to make good contact. The electroless copper plating technology can be used in the application of flexible electronics, where good conductive lines have to be fabricated on flexible substrates such as plastics or textiles, which usually cannot sustain high-temperature treatment.

**Fig. 4:**
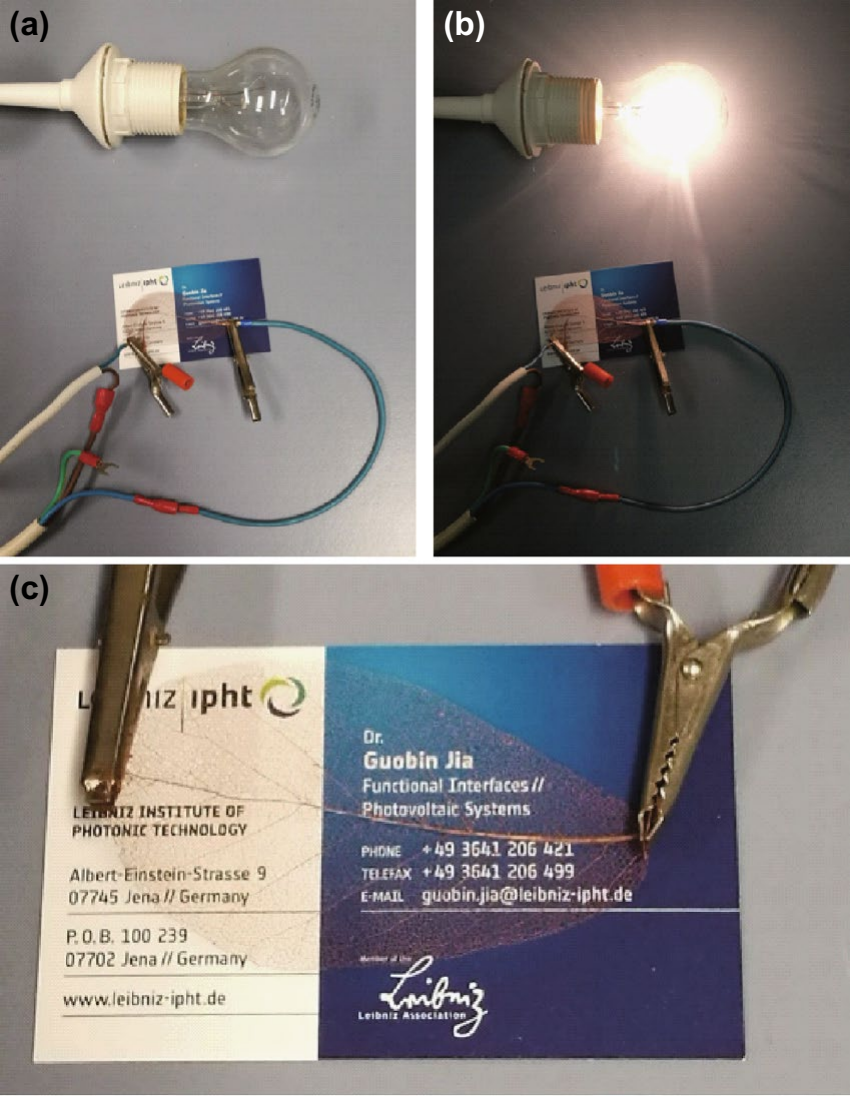
Demonstration of high current density transport capability. **a** The 30 min as-plated MLLV is connected between the cables. **b** A bulb (40 W and 230 V) shines if the switch is tuned on. **c** Photograph taken as the applied current of 1 A was passing through the Cu-plated vein

To visually demonstrate the high *J* transport capability and the high *T*, a 30 min as-plated MLLV is placed on top of a visiting card, through which a bulb (40 W & 230 V) is connected to the power supply system (50 Hz, 230 V), as shown in Fig. 4a. When switched on, the bulb shines (Fig. 4b). In this case, the current is 174 mA. We further used a current source to apply 1 A (Fig. 4c) through the same vein for at least 1 min. It performs very well, and no visible change or damage is observed.

### Long-Term Stability and Flexibility

Oxidation of copper is well known if it is exposed in air, and a thin film of copper oxides will form. To investigate the long-term influence of the oxidation to the sheet resistance, the *R*_sh_ of some of the Cu-plated MLLVs (5, 10, and 15 min) has been re-measured after one year (379 days), and their sheet resistances keep almost unchanged within the error range (Section S10). It is noted that these samples were stored individually in closed sample boxes (but not vacuum sealed) in my office.

The mechanisms of copper oxidation are very complex depending on the preparation methods, surface roughness, crystal orientations, surface contamination, as well as storage conditions, and a comprehensive review [44] to this topic was given by Gattinoni et al. One relevant study to ours has been presented by Keil et al. [45], who have studied long-term oxidation of copper thin films stored at ambient condition and observed finally a bilayer of 2.0 nm CuO over 3.5 nm Cu_2_O after a period of 9 months. Even with a linear extrapolation, the thickness of copper oxides should be less than 10 nm after one year, and this is far below those plated on the veins in our cases (> 500 nm), a ratio of only less than 2% with the oxides to the beneath copper layer can be followed, so that practically there is no observable influence to the electrical conductivity.

However, we do have observed increases in sheet resistance for the copper-plated MLLVs stored differently, i.e. sandwiched between two paper sheets. An increase in sheet resistance of 35% (20 min plated) and 31% (30 min plated) has been observed after nearly one year (355 days), respectively, and such results are rather unexpected since these samples have a much thicker copper layers of ~ 4 (20 min plated) and 5.5 µm (30 min plated), a less than 10 nm copper oxides cannot explain the large increase in the *R*_sh_ alone. However, in close view to the paper production, it is noticed that a lot of sulphur-containing chemicals are used, and the large increase in the *R*_sh_ might well be attributed to the sulphurization of the copper due to sulphur source in the paper.

For their application as electrodes for solar cells, they will be encapsulated anyway by ethylene vinyl acetate (EVA), sandwiched between two glass plates, and therefore will be isolated from air and any sulphur sources. An oxidation and sulphurization of the copper can be excluded, so that the long-term stability can be expected, but rather a great issue might be the copper-related deep-level defects discussed in Sect. 3.2, which degrades the performance of the solar cells, and the issue could be solved by a further metal coating such as Ag encapsulating.

For other applications, further solution for the anti-oxidation coating of the copper-plated vein-like electrodes can be done by electroplating of an encapsulating metal layer such as gold or nickel, and they can be easily realized on the conductive copper-plated networks by electroplating.

**Fig. 5:**
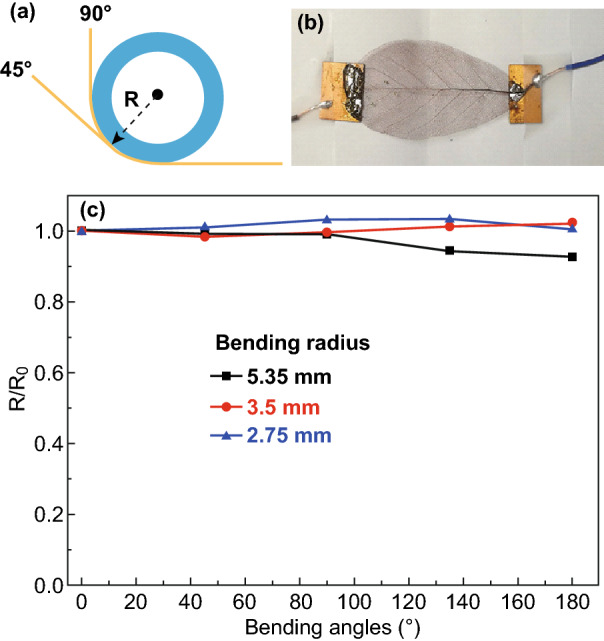
The image **a** illustrates the experimental set-up of the bending tests and **b** is a photograph of the sample soldered between two pieces of circuit boards and fixed on a piece of paper as mechanical support. **c** shows the normalized resistance (to the value measured at the bending angle of 0°) of a copper-plated MLLV measured during bending tests with different bending radii and angles

The copper-plated MLLV (30 min plated) has a remarkable low resistance around 0.35 Ω between the two solder positions. The results of the three bending radii show that the resistance keep almost constant within a fluctuation of 3.4% under all the bending angles, and this excellent stability of the resistance during the bending tests can be attributed to the very well ductility of the copper layer as well as the flexible vein network and are highly desired in flexible electronics (Fig. 5).

It is noted that the MLLVs still have many “redundant” free-hanging lower-order thin veins (Section S11). Obviously, they do not contribute to the measured *R*_sh_, but they do lower *T* and increase the Cu consumption for the Cu-plated veins. Nonetheless, the MLLVs demonstrate excellent ultralow *R*_sh_ combined with high broadband *T* and low Cu consumption. Furthermore, if such conductive networks can be prepared by 3D printing technology without the involvement of the leaf vein frameworks, the stems can be significantly thinned, and *T* can be further improved up to 95% with the same *R*_sh_. There is still plenty of room for improving the individual properties for specific applications. It is expected that such high-performance and aesthetic TCEs will open up many applications in building-integrated solar cells and in high-energy–density storage devices. In fundamental research, the TCE could be used in energy storage devices for in situ and even in operando [46] observation of the chemical and physical processes at the interface between the electrolyte and the electrode materials, in order to better understand the processes at the interface and to improve the properties of the devices.

It is demonstrated that the vein-like TCEs have low sheet resistance, high optical transparency, high current transport capability, and resource efficiency, and some of these properties may relevant for specific applications; for example, for solar cell applications, the high transparency, low sheet resistance, and low silver consumption may be the desired properties for mass production to increase the efficiency and reduce the cost. For Li-ion batteries, the low material consumption reduces the weight, allowing more active materials to be attached on the electrode, and this could be interesting for high power density storage batteries. For supercapacitor, the high current density transport properties will have advantages in high power density charging and discharging processes, in which a short pulse of high current density may involve.

Unlike in other biomimicry research, which often means that the same function is imitated from similar bio-structure, for example, nano- and microstructured surfaces have greatly contributed to the design of self-cleaning solar cells after the principle of the Lotus effect [47]. The widely used hook-and-loop fastener “Velcro straps” have been mimicked actually after the hooks on the plant seeds burrs [48]. The research demonstrated in this contribution is quite different, instead of mimic the material transport properties through the vein networks, the electrical transport through vein-like electrodes has been mimicked, and this may be a new idea for biomimicry. It is noted that the excellent transport function of the leaf vein architectures may be better understood by establishing mathematic model, which could help to solve similar questions related to bidirectional transport processes, such as street systems in large cities, optical fibre network data transfer systems, and power grids with separated power generation and consumption systems, and will provide design rules for the optimization of these complex systems.

The excellent transport property and high resource efficiency of the “naturally formed” vein networks arise the questions whether leaf vein networks formed by natural processes or intentionally creation and how the plants come to the superior architectures for ideal transport purposes.

## Conclusions

In summary, the electrical transport function of metallized vein networks has been mimicked from the material transport properties of the leaf vein networks in this contribution. It was demonstrated that the leaf veins can be converted to highly transparent conducting networks by electroless copper plating. The sheet resistance can be 2–3 orders of magnitude lower than that of the state-of-the-art ITO thin film, combined with a broadband (UV–VIS–IR) optical transparency up to 86%. In addition, the resource efficiency of the vein was demonstrated, which will inspire research in many fields related to replacing critical materials or reducing noble metal consumption for the electrode applications, which will be of great importance to a sustainable development, for example, in mass production of PV modules, and may significantly breakdown the cost, make PV competitive to conventional energy sources. The high current density transport capability of the vein-like electrodes is another great feature that will promote many applications, such as in high power density concentrator PV, high power density and light-weight Li-ion batteries and supercapacitors.

## Electronic supplementary material

Below is the link to the electronic supplementary material.

Supplementary material 1 (PDF 1677 kb)

Supplementary material 2 (MP4 5609 kb)

Supplementary material 3 (MP4 12836 kb)
