# Supplementary material for: Biomimic Vein-Like Transparent Conducting Electrodes with Low Sheet Resistance and Metal Consumption
Source: Nanomicro Lett. 2020 Jan 10;12:19. doi: 10.1007/s40820-019-0359-9 (PMC7770790; doi:10.1007/s40820-019-0359-9)
Supplement: Supplementary file 1 — Supplementary material 1 (PDF 1677 kb) [file 40820_2019_359_MOESM1_ESM.pdf]

Supporting Information for

Biomimic Vein-like Transparent Conducting Electrodes with Low Sheet Resistance and Metal Consumption

Guobin Jia[1, *], Jonathan Plentz[1], Andrea Dellith[1], Christa Schmidt[1], Jan Dellith[1], Gabriele Schmidl[1], Gudrun Andrä[1]

[1]Leibniz Institute of Photonic Technology (Leibniz IPHT), Dept. Functional Interfaces, Albert-Einstein-Str. 9, 07745 Jena, Germany

*Corresponding author. E-mail: guobin.jia@leibniz-ipht.de (Guobin Jia)

S1 Error Tolerance of the Leaf Veins

The leaf vein networks have been ideally optimized for the bi-directional transport function, ensuring the resources to be distributed and collected even if the leaves are damaged due to wormholes as shown in the examples in Fig. S1. However, the leaf cells around the damaged positions are not strongly influenced.

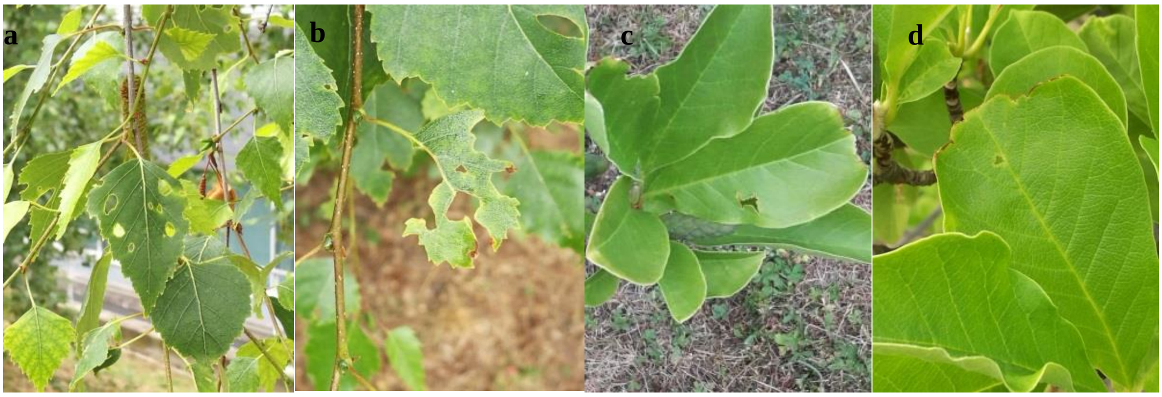

Fig. S1 Wormholes on *Betula pendula* (**a**, **b**) and *Magnolia liliiflora* (**c, d**) leaves

S2 Bi-directional Transport of Water through the Vein Network

Video S1 demonstrates the water transport from the leaf blade to the petiole.

Video S2 shows the water transport from the petiole to the leaf blade.

S3 Determination of the Area of the Veins

The areas of the veins were determined by drawing the contour of the vein onto a paper with known size (area). Then it was cut out of the paper, and the weight ratio is proportional to the area ratio of the cut piece, so that the area can be determined.

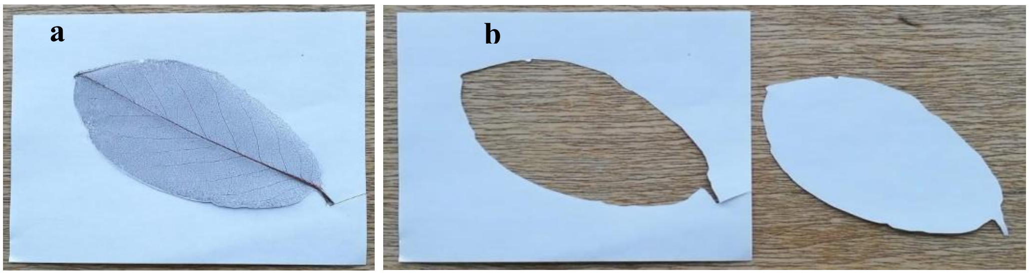

Fig. S2 a) Draw the contour of the leaf vein on a paper with known size. **b**) Cut the contour out of the paper and weigh both pieces of the paper. The area of the vein can be determined with the weight ratio.

S4 Thickness of the Cu Layer on the Vein

The thickness of the Cu layer is determined by SEM (left) images and the corresponding back scattered electron (BSE) images (right). In the latter case one can distinguish the metal from non-metal as shown in Fig. S3. Due to the irregular shape and rough surface of the veins, it is not possible to measure the thickness accurately. For the 5 and 30 min Cu-plated MLLVs, a thickness of ~0.5 and 5.5 μm can be determined from the BSE images, respectively.

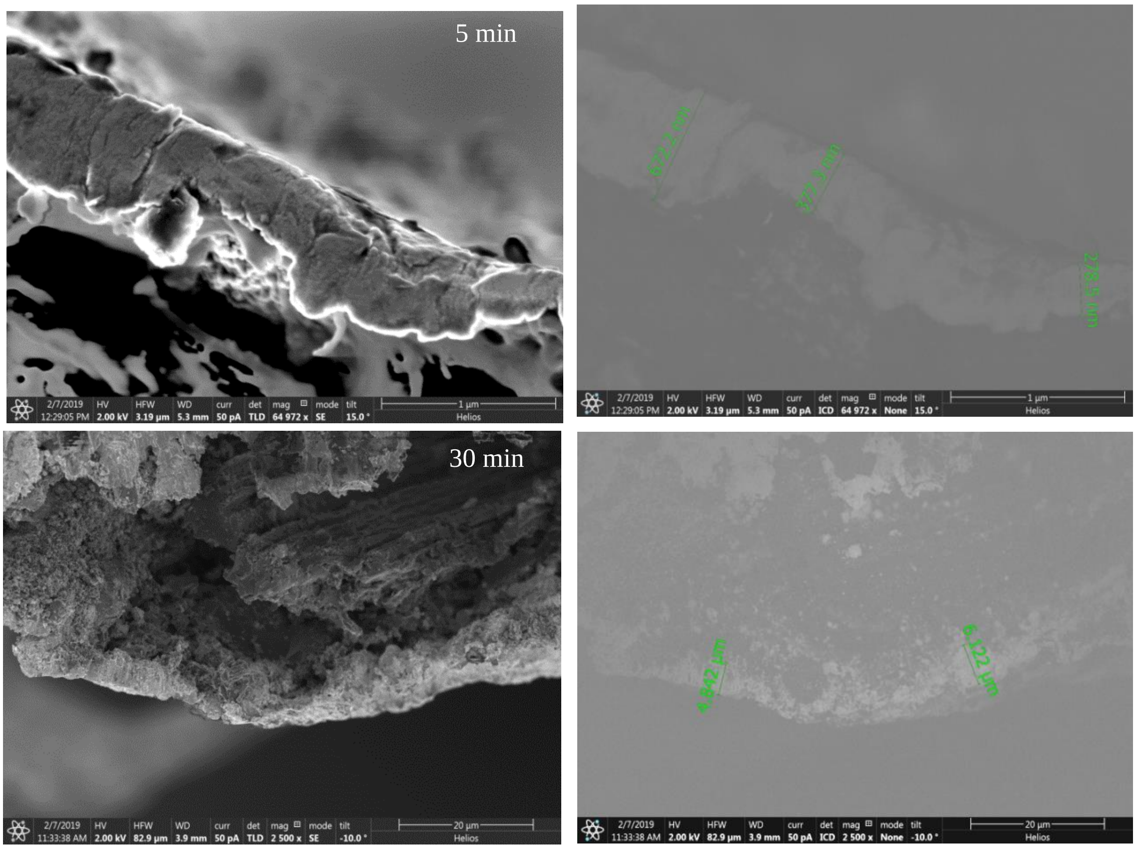

Fig. S3 Cross-sectional SEM (left) and the corresponding BSE images (right) for 5 min (upper) and 30 min (lower) as-plated MLLV.

S5 Annealing of the Veins and the Cu-coated Veins

As-prepared veins as well as 30 min Cu-plated veins (MLLV, Fagus sylvatica, and Populus × canescens) are placed on quartz glass substrates. They were placed in the tube furnace under

inert N_2. After 30 min, the furnace temperature was slowly raised (~8 °C min^{-1}.) in several steps:

- Keep at 100 °C for 20 min
- Keep at 150 °C for 30 min
- Keep at 200 °C for 40 min
- Keep at 300 °C for 25 min
- The temperature was raised to 970 °C close to the melting temperature of copper and kept for 120 min.
- The samples were cooled down to room temperature overnight with a continuous flow of N_2.

By this annealing procedure, the as-prepared vein can be carbonized to a conducting carbon network (Fig. S4a). A resistance of ~1000 Ω has been measured on a carbonized MLLV using a multimeter at two points between a distance of ~2 cm. However, the carbonized network is very brittle and breakes easily during the four-point probe measurement, and so do not allow for systematic measurements. Anyway, we compared similar measurements between two points on the annealed Cu-plated veins with known R_{sh}, and estimate that of the carbon network to be at around 100 $\Omega\ \square^{-1}$.

Surprisingly, with the Cu-plated veins, mechanically stable networks can be obtained after the annealing. This thermal treatment leads to nearly half of the R_{sh} down to an ultralow value of 68 m$\Omega\ \square^{-1}$ with T=85% with the 30 min plated MLLV. After the annealing, a schrinkage of the vein area down to ~55% can be determined as shown in the examples in Fig. S4b for the 30 min Cu-plated *Populus* × *canescens* leaf vein on top of a quartz substrate (2.5×2.5 cm^2) before (b) and after (c) the thermal annealing procedure in Fig. S4c.

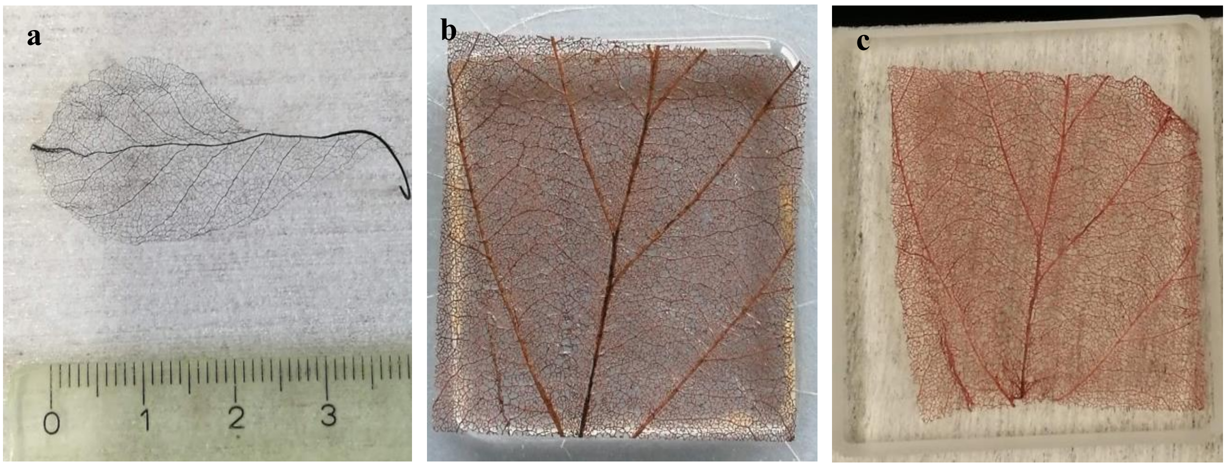

Fig. S4 Thermal annealing of the vein and Cu-plated veins. **a**) A piece of carbonized (at temperature up to 970 °C) MLLV. **b**) A piece of 30 min Cu-plated *Populus* × *canescens* leaf vein on top of a quartz substrate (2.5×2.5 cm^2) before (**b**) and after (**c**) the thermal annealing

S6 Results Obtained from *Fagus Sylvatica* Leaf Veins with Higher Vein Density

The Fagus sylvatica leaf veins (FSLV) have higher vein densities as shown in comparison with MLLV of Fig. S5a. The 30 min Cu-plated FSLV reaches a R_{sh} of 0.038 $\Omega\ \square^{-1}$with a T=75.2% (value at 550 nm). After the annealing, an exceptionally low R_{sh} of 0.018 $\Omega\ \square^{-1}$ with decreased T=61.8% has been reached (see Fig. S5b).

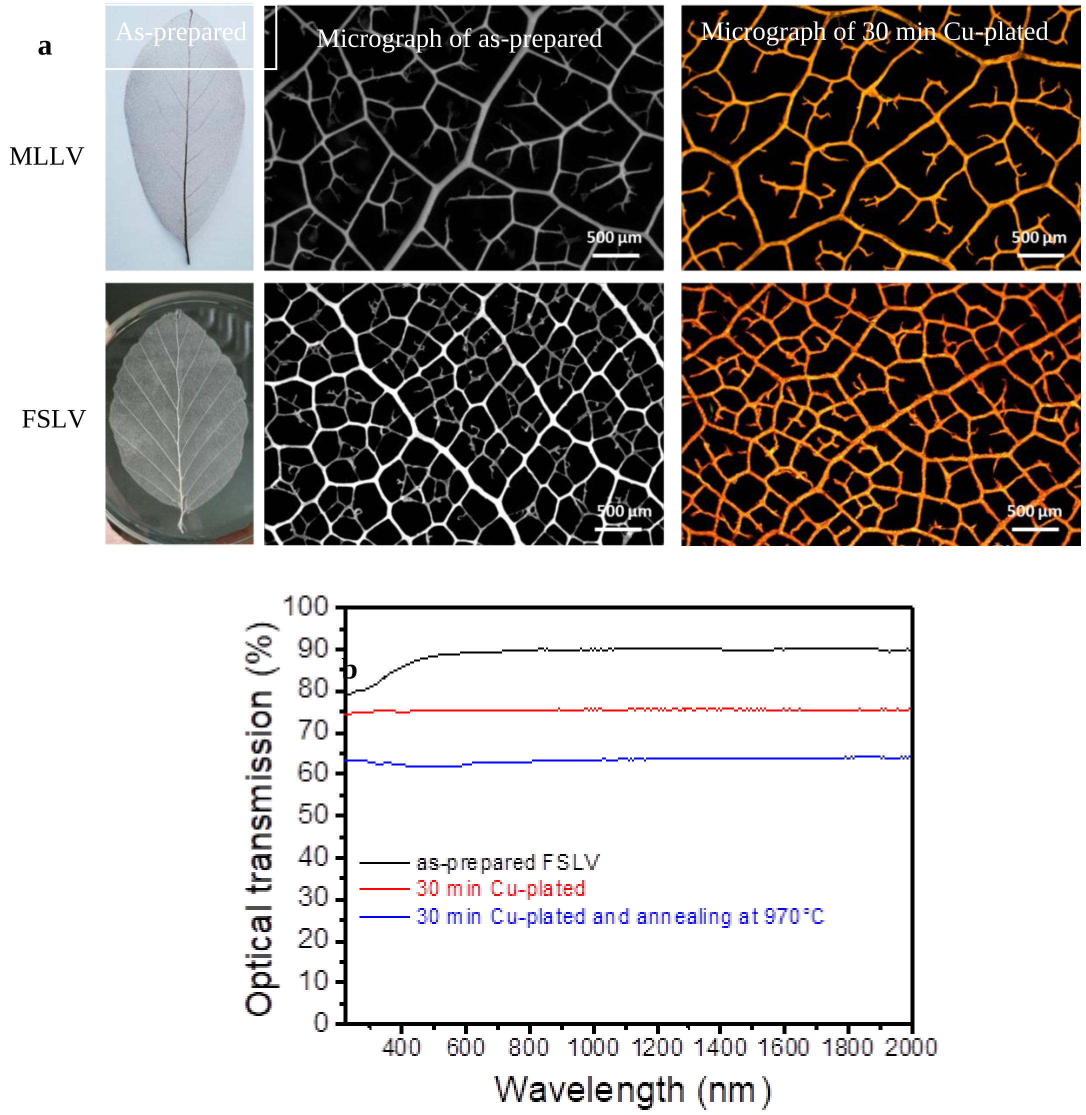

Fig. S5 a) Comparison of the MLLV with the dense FSLV in micrographs. **b**) The optical transmissions of as-prepared FSLV (black line), 30 min Cu-plated (red line) and that after the annealing (blue line).

S7 Surface Morphology

Even after a short plating time of 5 min, a thin layer of copper covering completely the vein network can be seen in the SEM images (Fig. S6).

Cu crystallites are grown on the veins during the ECP forming a compact layer during the ECP even within 5 min. The average crystal sizes of the as-plated samples are in the range of 45-75 nm determined by X-ray diffraction (XRD). A substantial improvement of the crystallinity and growth of larger crystallites up to 140 nm has been determined after the thermal annealing. This can be confirmed by the SEM images in Figs. S7 and S8. The sample annealed at 970 °C show large crystals with clear facets. The improved crystallinity and grown of larger crystals could explain the decrease of the R_{sh} upon annealing.

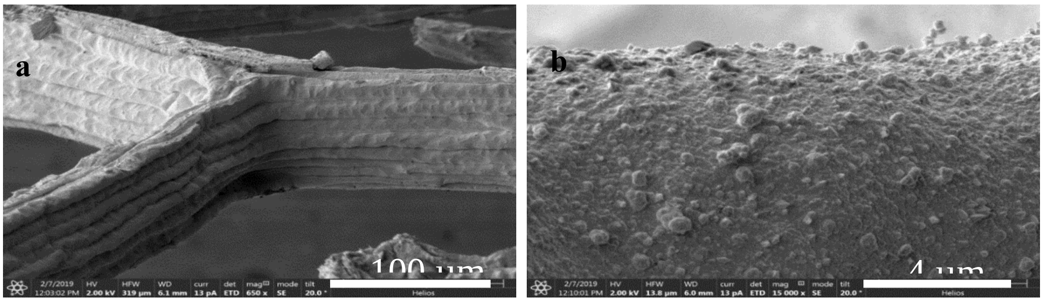

Fig. S6 a) SEM image of the 5 min as-plated MLLV. **b)** High resolution SEM image showing a compact layer of Cu crystallites.

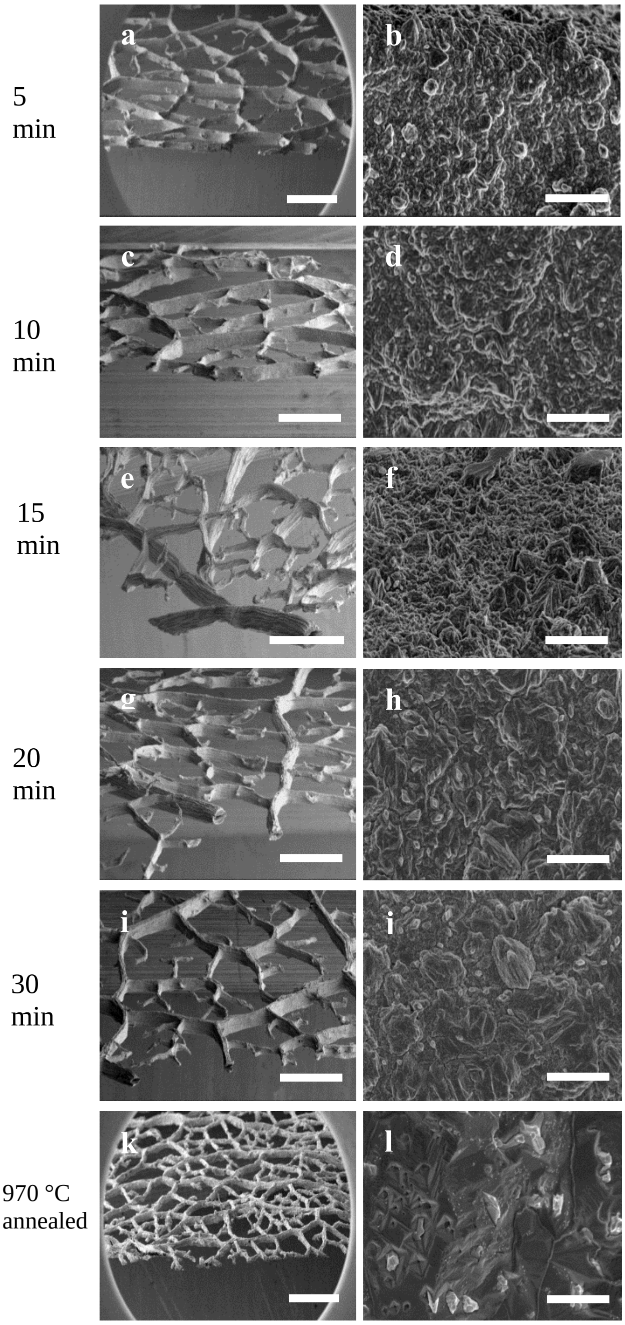

Fig. S7 SEM overview (left, scale bars: 500 µm) and the corresponding high resolution (right, scale bars: 1 µm) SEM images of the as-plated MLLVs with different plating times and those after the annealing procedure in **k** and **l**

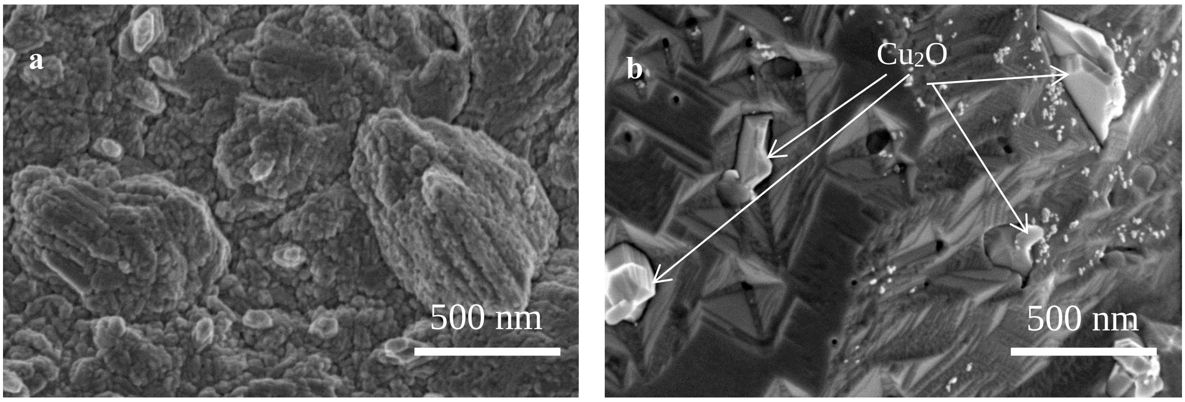

Fig. S8 Comparison of the morphology of the as-plated vein and that after the annealing procedure. **a**) High resolution SEM image of typical as-plated MLLV (30 min plated). **b**) High resolution SEM image of annealed MLLV showing large crystallites and clear facets on the surface. A small amount of cuprite (Cu_2O) crystallites are present on the annealed sample.

S8 Estimation of Current Density

The contact area of the tip is 0.194 mm^2 (diameter of 0.88 mm) (Fig. S9a), so a J of 51.54 A cm^{-2} passing locally through the two outer tips can be determined. Considering each tip is usually in contact with 6 to 10 of the thin veins as illustrated in Figure S9b, each of them can sustain at least 10 mA. From the cross-sectional SEM and the corresponding backscattered electron images (see S6), the thickness of the Cu layer can be estimated to be about 0.5 and 5.5 µm for the 5 min and 30 min plated samples, respectively.

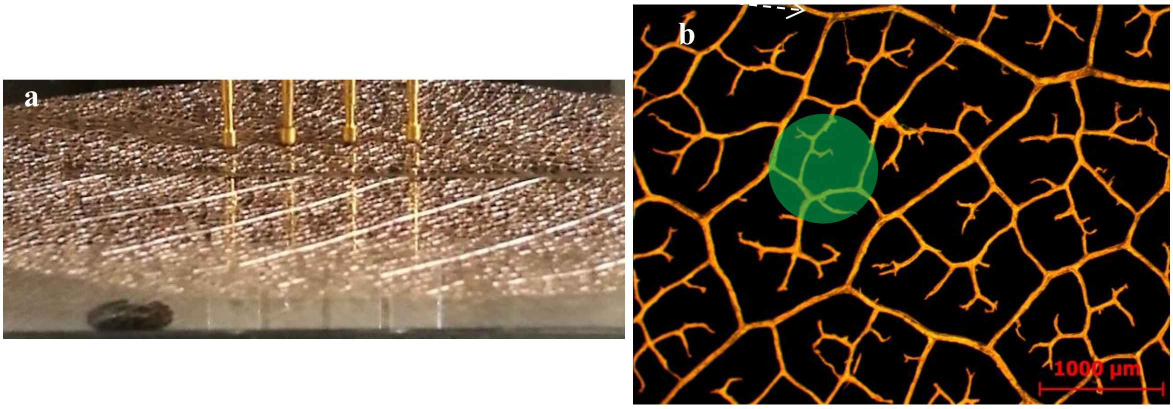

Fig. S9 a) A photo showing the as-plated MLLV during the four-probe measurement. **b)** The tips of the four-point probe measurement contacts on the MLLV. Six to ten thin veins could be contacted by the tips with a diameter of 0.88 mm

Figure S10a, b is SEM image and a sketch of the cross-section of the 5 min plated MLLV, respectively. For a conservative estimation of the cross-sectional area of the copper-plated veins, a circle with an equivalent diameter of 100 µm of the thin veins is used, considering the rough surface of the veins (Figure S10c), and the cross-sections of the 0.5 µm copper layer plated on the 100 µm veins can be calculated to be $\pi(50+0.5)^2-\pi(50)^2\approx158$ µm^2, and that for the 5.5 µm copper layer is 1823 µm^2 (see Figure S10d and S10e). So current densities of 6329 (5 min plated) and 549 A cm^{-2} (30 min plated) passing through the cross-section of the copper layers can be calculated using the 10 mA for each of the thin veins, respectively (noting that a high efficiency solar cell generates a J of only ~40 mA cm^{-2}).

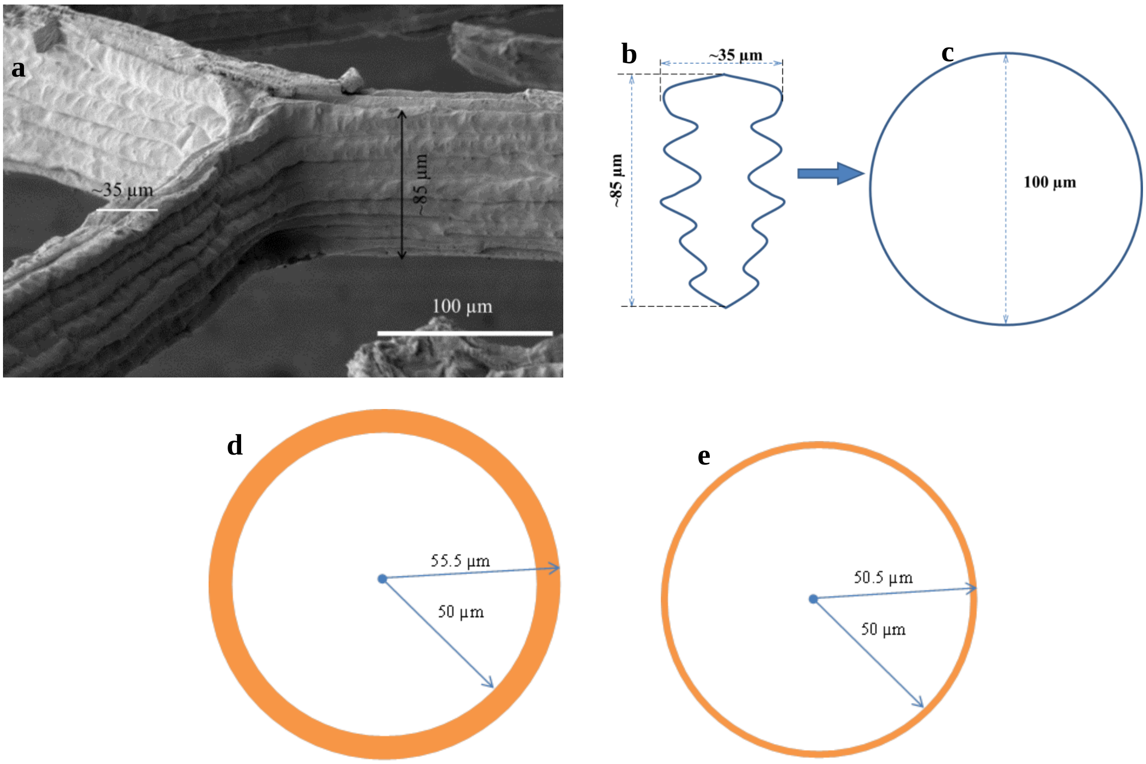

Fig. S10 a) SEM image of the 5 min plated vein. **b**) Sketch of the cross section of the vein. **c**) A circle of 100 µm as the equivalence of the cross section of the vein. **d**, **e** Sketches for the calculation of the cross-section of the copper layers with thicknesses of 0.5 (**d**) and 5.5 µm (**e**)

S9 Easy Handling with the Veins and Solderable on a Circuit Board

The Cu-plated veins can be easily handled and even soldered onto a circuit board as shown in the image of Fig. S11a and the micrograph of Fig. S11b. The contact is mechanically stable and gives a very good ohmic conductivity.

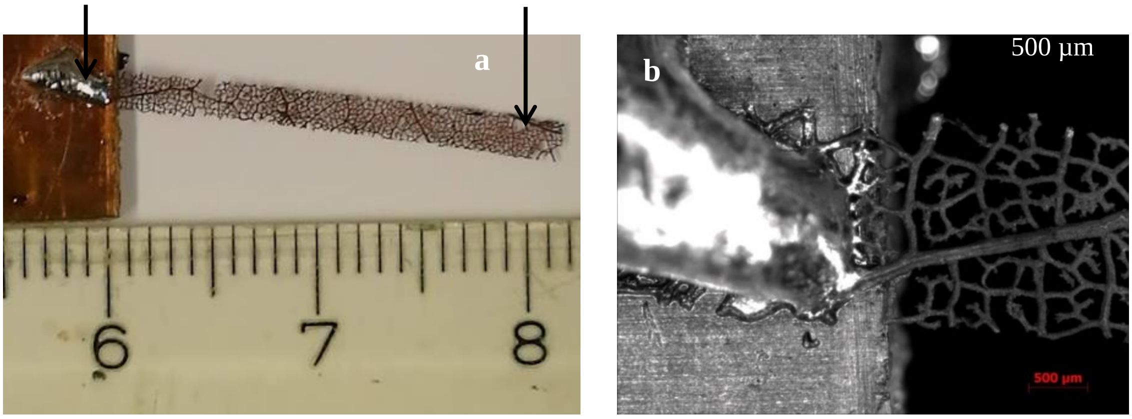

Fig. S11 a) A stripe of a 30 min Cu-plated MLLV soldered (at ~350 °C) on a circuit board. A resistance of 0.8 Ω is measured between the two arrows. **b)** The micrographes of the soldered contact.

S10 Long Term Stability

Oxidation of copper is well-known if it is exposed in air, a thin film of copper oxides will form. To investigate the long-term influence of the oxidation to the sheet resistance, the R_{sh} of

some of the Cu-plated MLLVs (5, 10, and 15 min) has been re-measured after one year (379 days), and their sheet resistances keep almost unchanged within the error range. It is noted that these samples were stored individually in closed sample boxes (but not vacuum sealed) in my office.

However, we do have observed increases of sheet resistance for the copper-plated MLLVs stored differently, i. e. sandwiched between two paper sheets. An increase of sheet resistance of 35% (20 min plated) and 31% (30 min plated) have been observed after nearly one year (355 days), respectively, and such results are rather unexpected since these samples have a much thicker copper layers of ~4 (20 min plated) and 5.5 µm (30 min plated), a less than 10 nm copper oxides cannot explain the large increase of the R_{sh} alone. However, in close view to the paper production, it is noticed that a lot of sulfur-containing chemicals are used, and the large increase of the R_{sh} might well be attributed to the sulfurization of the copper due to sulfur source in the paper.

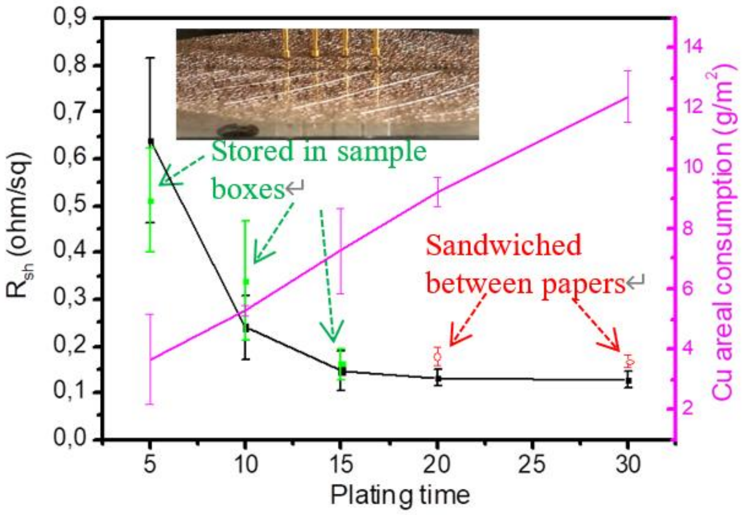

Fig. S12 The green squares with error bars are copper-plated MLLVs measured after one year (379 days), and the samples were stored individually in sample boxes, and the red circles are those measured after (355 days), which were sandwiched between two paper sheets.

S11 "Redundancy" of the MLLVs

It is noted that the MLLVs have still many "redundant" free-hanging lower-order thin veins as demonstrated in Fig. S13a. The "redundant" free-hanging lower-order veins have been removed via image processing in Fig. 13b. Obviously, the free-hanging thin veins do not contributed to the measured R_{sh} since no current can pass through them. They are detrimental to the T as well as to the Cu consumption for the Cu-plated veins. Nonetheless, the MLLVs demonstrate the excellent ultralow R_{sh} combined with high broadband T and low Cu consumption.

Furthermore, the vein networks are only used as framework for the growth of the Cu crystallites on them. If the same amount of copper can be deposited directly onto devices without the framework, for example, by direct 3D printing technology, a greatly reduced thickness of the stems may reach $R_{sh} << 0.1\ \Omega\ \square^{-1}$ with a broadband T up to 95% .

There is still plenty of room for improving the individual properties for specific applications.

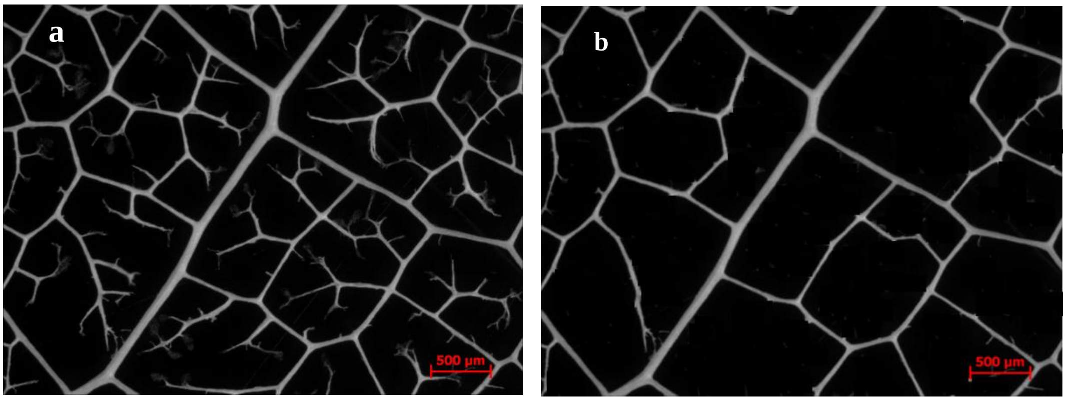

Fig. S13 a) Micrograph of a *Magnolia liliiflora* leaf vein in original state. **b)** Removal of the “redundant” free-hanging lower-order thin veins via image processing.
